# Peer effects among friends on students’ cognitive abilities: An analysis based on emotional distance

**DOI:** 10.1371/journal.pone.0312190

**Published:** 2025-02-03

**Authors:** Wenyi Zhou, Junming Xie, Jinsong Kuang, Yongqing Feng, Dag Øivind Madsen

**Affiliations:** 1 XiangNan University School of Economics and Management, Chenzhou, Hunan, China; 2 College of Economics and Trade, Hunan University of Technology and Business, Changsha, Hunan, China; 3 Guizhou University of Finance and Economics, Guiyang, Guizhou, China; 4 Davis School of Business, Colorado Mesa University, Grand Junction, Colorado, United States of America; Programa de Pós-Graduação em Administracao da Universidade Paulista (PPGA-UNIP), BRAZIL

## Abstract

In contemporary society, students’ cognitive abilities are crucial for the accumulation of human capital. Consequently, significant concern has been expressed regarding the impact of peer effects among friends on students’ cognitive abilities. Based on the "China Education Tracking Survey" data, we discuss peer effects among friends on students’ cognitive abilities from the perspective of Emotional Distance Analysis. Our study shows that: (1) Students’ average scores could be actively affected by the increase in the number of friends with good grades. (2) Peer effects among friends are in accord with different students. Such effects are more easily exerted among female students and those with better average grades or local household registration. (3) The mechanism study found that the conduction effect of peer effects among friends mainly relies on two paths. One is manifested as the “compliance effect”. In other words, to align with their friends, students increase their study time, raise their educational aspirations, and minimize instances of skipping classes and absenteeism. The other channel is the “anchoring effect,” which involves raising parents’ reference standards, leading them to devote more time and energy to their children’s learning. Therefore, the rational use of social interaction with friends is a vital approach to enhancing students’ cognitive abilities.

## 1 Introduction

As one of the manifestations of social interaction, peer effects have a crucial impact on cultivating students’ cognitive abilities. In recent years, peer effects have been extensively studied and applied in the field of health [1, 2], consumption [3, 4], choices related to university majors or careers [5, 6], and corporate governance [7, 8], to name just a few examples of research areas. Moreover, peer effects have been a longstanding area of interest within economics and other social sciences [9]. Therefore, understanding and assessing peer effects is key to studying social issues [10]. Meanwhile, peer effects play an important role in the development of students’ cognitive abilities. Thus, based on the China Education Tracking Survey (CEPS) data, we utilize regression analysis to explore how the number of close friends with high academic achievements affects students’ academic performance. This approach offers a novel research angle for enhancing students’ cognitive abilities.

According to previous research, students’ academic performance is generally used to measure their cognitive abilities [11]. Academic success is closely related to educational input, in which peer input is an important component [12, 13]. In the famous Coleman Report, [14] identified peer input as a major challenge for disadvantaged students. Subsequently, some studies found that peer interaction was one of the core factors affecting academic performance [10]. Influ enced by their peers, minority students may intentionally set lower academic aspirations to align with the norms of their group [15].

While students’ academic performance is affected mainly by individual efforts, it is also influenced by peers, such as classmates or friends. Some researchers view the peer role as equally important as other inputs (teacher quality and parental involvement), a key factor in students’ academic achievement [16]. However, it is difficult to identify the magnitude of peer effects. This is because the magnitude of peer effects depends on the frequency of group interactions, which is heavily impacted by the emotional and physical distance between members. Compared with geographical distance, the emotional one is more important, because the networks formed among friends tend to have a more stable and enduring impact than temporarily formed spatial peer relationships [17].

This study emphasizes peer effects stemming from the emotional closeness among friends, as individuals within similar groups tend to interact more frequently with each other, thereby affecting each other’s cognitive behaviors. Although such interactions may intensify the issue related to self-choice, they could boost students’ intrinsic drive and individual performance [6]. (Note: The CEPS survey shows that when students want to chat with someone, the vast majority (83%) will first think of their good friends. When students want to ask for help, most (56%) will first think of their good friends, far exceeding the proportion of students who first think of their parents. Therefore, the peer effect among friends is quite important for students. Second, in terms of measurement methods, compared with the usual linear mean model, it could be more intuitive and scientific to measure peer effects by the proportion of friends with good grades.

The rest of the paper is structured as follows: The second part provides a literature review and outlines research hypotheses. The core research hypotheses are formulated after an extensive examination of existing literature. The third part briefly introduces the data sources, the main variables used in the empirical model, and descriptive statistics. The fourth part presents empirical results and analyses. To address any possible endogeneity issues, benchmark regression is first performed on the model, and the heterogeneity analysis is then conducted based on average grade, household registration, and gender. The fifth part analyzes the transmission mechanism of the influence of the number of friends with good grades on students’ academic performance and carries out the robustness test. The sixth part draws the main conclusions.

## 2 Literature review and research hypotheses

### 2.1 Peer effects among friends and students’ cognitive abilities

As part of social networks, each independent individual could be inevitably impacted by others’ decisions and behaviors. Middle school students, whose minds and thoughts are still developing, are more susceptible to the influence of their peers, such as classmates and friends. Parents strive to ensure that their children can attend good schools to secure a beneficial learning environment. Peers, such as classmates and friends, have become an important part of this environment.

Existing studies on peer effects primarily concentrate on how students’ grades are affected by such peers as classmates, friends, and neighbors. However, it is not clear whether peers significantly improve students’ academic performance [18]. Several studies suggest that the peer effects could significantly improve students’ academic performance. For instance, [19] examined the effects of roommates’ educational backgrounds on each other’s academic performance through a quasi-natural experiment of randomly assigning roommates. The findings indicated that roommates’ academic performance would significantly affect that of the other students. According to [20] research, students’ academic performance could improve with an increase in peer quality. [21] study indicated that every standard deviation rise in peers’ SAT scores could bring about a 0.05 standard deviation increase in other students’ GPAs. Similar findings have been made by [22–24]. Meanwhile, numerous studies have also found that peer grades have little substantial impact on students’ grades. In a study based on the Metco Project in Boston, [25] discovered no overall peer effect. [26] found in their study on local school performance after Hurricane Katrina that the influx of evacuated students had just a slight linear average effect on students’ academic performance. In their study on the relationship between students’ performance and peers in classrooms ranging from Grade 3 to Grade 10, [27] concluded that the peer effect is small or even non-existent. Similar conclusions were reached by [28–31]. [31] even demonstrated that it is significantly negative for the class average’s overall impact on academic performance.

Faced with these two contrasting conclusions, one explanation is that applying a simple linear mean model would underestimate the impact of the peer effect [10], as researchers may have potentially presumed that it would be homogeneous under the influence of group behavior on all members [32]. Therefore, the estimated peer effect could be larger and more significant when relaxing the linear mean assumption and adopting nonlinear models [20, 25, 27, 29, 31] even though different scholars hold different views on which students benefit more from such an effect [21, 24, 26].

The other explanation could be that earlier studies on peer effects were predicated on the idea that the individual has the same effect on others in the same group. It is hard to assume that all members in a large group have the same degree of intimacy or geographical distance from one another. This is because the peer effect could be limited by the frequency of social interaction among members [33], which would be also determined by the degree of intimacy and geographical distance between individuals. Even if individuals within the same group have different influences on others due to differences in intimacy, individuals with closer relationships have a greater impact on themselves, and vice versa [32]. At the same time, there might be subgroups within the group, and only members of the subgroup could have sufficient social interaction with each other. For example, [34] found it easy to form small groups within a class and that individual abilities could be affected by peer effects generated through social interaction in such groups. Therefore, estimating the impact of group means on individuals in a general way can lead to an underestimation of the peer effect. As the group size increases, identifying the peer effect can become more challenging because the less significant influences of other members may obscure the more substantial impacts within the subgroup [35]. [27] found that the peer effect was significantly greater at the class level than at the grade level, as the latter was not significant in most cases. When discussing the peer effect in a class, few researchers believe that it stems from the influence of class average performance on individuals. In contrast, the peer effect observed often comes from the influence of one friend or a group of close friends [10].

Therefore, it is not enough to control the fixed effects only at the school or class level since it can lead to the neglect of group characteristics among student groups within the class, resulting in serious endogeneity issues [36]. Our solution is to narrow the scope of the group and explore the influence of the social interaction between the best friends of middle school students on their academic performance. The reason for choosing to study the peer effect among friends in middle school is that students in that age group are beginning to break away from family but are still not completely independent. The influence of parents remains present, albeit in a diminishing capacity [37], and students’ behaviors are largely influenced by peers in daily life and learning [13]. Besides, internal consistency can be ensured, as the circle of friends in middle school is relatively small but the frequency of their social interaction is higher. And there has been plenty of previous research on the peer effect of test and non-test scores among friends. For example, [38] found in experiments that the frequency of going to the gym significantly increased when subjects had more friends who liked working out. Conversely, when an individual has friends who become obese, their probability of becoming obese also increases substantially [1, 39]. The peer effect could be diminished when taking into account the background characteristics of friends [40]. Several studies suggest it is highly likely that teenagers will participate in the same activities as their friends, including engaging in detrimental behaviors such as smoking, drinking, and skipping classes [37]. [41] conducted an experiment on the training of female entrepreneurs in India, which showed that the effectiveness of training could be significantly improved if women entrepreneurs attended a training session with friends. A study on adolescent friendship by [42] shows that there is a strong and lasting peer effect in education, but only those relationships that last for more than one year are influential. [43] showed that the total academic performance of a class with 20 students could be reduced by 2% of the standard deviation after a new student experienced local violence and joined a school in an area not directly affected by violence.

Compared with previous studies that focused on the influence of geographical distance such as the same dormitory or adjacent seats [19, 36, 44], we focus on the peer effects generated by emotional distance under the premise of a similar geographical distance (81% of the five best friends of students in the sample are classmates from the same school, and over 65% are classmates from the same class). Unlike the class selection system in Western schools, the daily teaching activities in Chinese primary and secondary schools are mostly carried out in fixed classes [45], leading to a high frequency of interaction among friends. However, social interaction among friends can transmit social norms, values, and learning skills. Therefore, it can have a significant impact on individual learning attitudes, cognitive abilities, and academic performance [46]. Therefore, the peer effects among friends may have a greater impact on students’ academic performance.

**Hypothesis 1: The more friends with good grades a student has, the better their academic performance.**

### 2.2 Mechanism 1: Peer effects among friends, family environment, and students’ cognitive abilities

Since the vast majority of students have their best friends in the same school or even class, it ensures enough social interactions among friends but also makes it difficult to identify the peer effects among friends. This occurs because the schools and classes students enroll in and the friendships they form are not random outcomes but rather the result of their parents’ considerable time and financial investment [10]. In addition, children’s choice of friends is also largely influenced by parents’ attitudes towards their friends. Therefore, when parents limit their children’s choice of friends, they tend to choose students with good grades as friends out of a desire for children’s success. In some extreme cases, parents may pay more to enroll their children in better schools to protect them from the bad behavior of peers. For example, many parents competing for their children’s admission into prestigious schools only aimed to provide a good learning environment for children, just as in the story of "Mencius’ mother moved three times" in China. (Note: Mencius’ mother moved many times and did all she could to provide the best environment for her child.).

Children might make friends based on their interests and aspirations when parents do not care about the state of their children’s friendships. However, since students with similar grades may have the same interests and hobbies, there may still be a problem of self-selection, i.e., students with good grades make friends with each other. Students’ choice of making friends can be partly random if they restrict their interests and hobbies (e.g., non-academic hobbies such as music, dance, and sports).

**Hypothesis 2: The more parents care about their children’s choice of friends, the more likely their children are to make friends with good grades.**

### 2.3 Mechanism 2: Peer effects among friends, anchoring effect, and students’ cognitive abilities

The existing studies on peer effects should deepen the understanding of the effect’s transmission mechanism, which is important for identifying problems and conveying information about human behavior [47]. The transmission mechanism of the peer effects among friends mainly includes the transmission of information and skills, sharing of knowledge, and changes in preferences and behaviors [33], in which the latter stems from compliance with group norms and the imitation of other members’ behaviors.

Students in adolescence usually lack the ability to think independently, and they hope to be recognized, admired, and respected by peers during their interactions [45]. Thus, they tend to imitate and compete with each other. The academic perceptions and performance of friends can change that of individuals, thereby affecting their engagement in learning. Consequently, the group’s recognition of learning could be higher as there is an increasing number of friends with good grades. To meet the changes in the learning atmosphere among groups, students would improve their educational expectations and spend more time on learning rather than entertainment, with less class truancy and absenteeism.

Besides, parents’ evaluation of their children’s academic performance could be influenced by their children’s academic performance and by the academic performance of their children’s friends. Friends with good grades could become role models in school and serve as reference standards for parents to evaluate their children’s performance. Therefore, when the number of children’s friends with good grades increases, it will not only affect children’s values and preferences but also indirectly improve parents’ educational expectations and performance requirements for their children, thereby increasing their educational input. For instance, increased parental concern for their children’s academic performance, dedicating more time to tutoring, and regularly reviewing homework can all contribute to enhancing their children’s academic achievements.

**Hypothesis 3a: The greater the number of friends with good grades, the higher a student’s educational expectations and the more time and attention they devote to their studies.**
**Hypothesis 3b: The greater the number of friends with good grades, the higher parents’ educational expectations and requirements for their children, and the more they will invest in their children’s education.**

## 3 Model setting and variable descriptions

### 3.1 Model setting

Drawing on the model selection by [10], a basic linear regression model was first constructed to estimate the influence of the number of friends with good grades on a student’s academic performance. The estimation equation of the linear mean value is shown as follows:

$${{\text{Y}}^{\text{j}}}_{\text{ics}}=\alpha_{0}+\alpha_{1}{\text{FP}}_{\text{ics}}+\alpha_{2}{\text{M}}_{\text{ics}}+\beta_{\text{c}}+\gamma_{\text{f}}+{\text{u}}_{\text{ics}}$$ (1)

In the model, "i" represents the student individual, "c" represents the class, "s" represents the school, "j" represents the three subjects of Chinese, mathematics, English, and their averages, and "Y" is the explained variable, representing the student’s scores. "FP", the core explanatory variable, represents the number of those with good grades among five best friends of students. "β_c_" represents the fixed effect of class, and "γ_f_" represents the fixed effect of friends.

"M" represents control variables, mainly encompassing two aspects. The first pertains to personal characteristics, including gender, household registration, age, cognitive abilities, only child status, number of siblings, living with parents, self-rated health status, and whether the individual has visited the hospital for medical treatment in the past six months. The second aspect concerns family characteristics, such as parents’ years of schooling, parents’ political status, parents’ occupation, relationship with parents, family economic status, number of books in the household, and access to computers and the internet. The descriptive statistical results of the main variables in this study are shown in Table 1.

**Table 1:** Descriptive statistics of major variables.

Variables	Observed value	Mean value	Standard deviation
Chinese score	17970	70.05	9.849
Mathematics score	17958	70.03	9.886
English score	17962	70.05	9.889
Average score	17916	70.07	8.601
Number of friends with good grades	18321	2.58	1.398
Age	18048	13.52	1.242
Gender	18168	0.52	0.500
Local registered residence	18107	0.82	0.384
Registered residence characteristics	17554	0.31	0.463
Ethnic group	18382	0.91	0.282
Only child or not	18422	0.43	0.495
Number of siblings	15706	0.64	1.020
Living with their mother or not	18154	0.86	0.344
Living with their father or not	18154	0.81	0.396
Self-rated health status	18274	4.06	0.895
Have been for medical treatment in the past six months or not	18291	0.08	0.274
Students’ educational expectations	18302	16.30	3.489
Study and entertainment time ratio	13966	0.79	1.470
Frequency of Class truancy	18302	1.09	0.431
Mother’s years of schooling	18091	9.52	3.548
Father’s years of schooling	18055	10.30	3.130
Maternal occupation			
Farmers, self-employed and unemployed individuals	16971	0.55	0.497
Ordinary staff and workers	16971	0.26	0.440
White-collar workers	16971	0.10	0.303
Corporate executives, party and government officials, and leaders of public institutions	16971	0.08	0.278
Paternal occupation			
Farmers, self-employed and unemployed individuals	17047	0.45	0.498
Ordinary staff and workers	17047	0.17	0.374
White-collar workers	17047	0.25	0.432
Corporate executives, party and government officials, and leaders of public institutions	17047	0.13	0.337
Mother’s political status	18427	0.04	0.195
Father’s political status	18427	0.06	0.231
Relationship with parents	17991	0.84	0.369
Family economic status	17681	2.99	0.555
Number of books in the family	18377	3.15	1.207
Status of computers and networks	18205	1.29	0.911
Parents’ expectation for their children’s education	18289	15.81	3.205
Parents’ academic requirements for their children	18324	2.83	0.906
Frequency of checking homework	18238	2.40	1.175
Frequency of tutoring	18065	2.02	1.112

Data Source: the 2013–2014 China Education Tracking Survey.

### 3.2 Description of variables

#### 3.2.1 Explanatory variable

The core explanatory variable in this study is the number of those with good grades among the five best friends of students. Based on the question, "How many of your best friends have achieved good marks?", in the CEPS student questionnaire, the answers are assigned as follows: assign 0 to the answer "none" and the mean value of 1.5 to the answer "one or two"; Since the CEPS questionnaire targets the five best friends of students, it is not difficult to draw a conclusion that the value should be 3 to 5 and the mean be 4 when the student answers "many". Thus, the assumption of the mean value will be relaxed in the robustness test of the paper.

However, there are still two exceptions: First, there is a difference in the proportion of friends with excellent grades when students have fewer than five best friends compared to those with more than five best friends, with each person accounting for more than 20% in the former case. The correction method we adopted is dividing the number of best friends with good grades by the number of best friends, then multiplying by five. As a result, every additional one in the number of students with good grades among all their friends represents a 20% increase in the proportion of friends with good grades. Second, the number of students’ best friends is less than the number of those with good grades among their best friends in some extreme cases. For the self-contradictory samples in the report (some of which the part is larger than the whole), we assigned the number of best friends to that of friends with good grades for descriptive statistics and regression analysis. Then, we deleted this part of the sample and conducted descriptive statistics and regression analysis again. It was found that there was no significant difference in the sign and significance of the regression coefficients when the conclusions drawn from these two sets of data were compared. Therefore, samples with smaller regression coefficients were selected to ensure the robustness of the conclusions.

#### 3.2.2 Explained variable

The explained variable used in this study is student performance. According to the CEPS Student questionnaire, student performance includes two levels of measurement: one is the original scores of the 2013–2014 fall mid-term exam in Chinese, English, and mathematics; The second is the standardized scores of these three subjects in schools and grades, with a mean value of 70 and a standard deviation of 10. In the CEPS questionnaire, the mean value was subtracted from the original score, then divided by the standard deviation, multiplied by 10, and added 70 to obtain the standardized scores with a mean value of 70 and a standard deviation of 10. There may be a slight difference between the mean and standard deviation obtained. To make the scores of students from different schools and grades comparable, we used the standardized scores in CEPS as the main indicator for measurement by drawing on [31, 48], and calculated the standardized average scores based on the score of each subject.

#### 3.2.3 Control variables

In terms of students’ personal characteristics, we used standardized scores from individual cognitive abilities tests and self-rated health status to control personal abilities and health levels, drawn on the selection of [48, 49]. Furthermore, these personal characteristics were accounted for as control variables in the analysis, i.e., the gender (boy = 1, girl = 0), age, household registration characteristics (urban = 1, rural = 0), household registration territory (county (district) = 1, others = 0), ethnic group (Han Chinese = 1, ethnic minorities = 0), the only child or not (only child = 1, non-only child = 0), the number of siblings, and living with parents or not (living together = 1, living differently = 0). Meanwhile, to reduce the subjective view of personal evaluation of health status, the dummy variable was added to fully describe their health status by whether an individual has been to the hospital for medical treatment in the past six months (have been = 1, have not been = 0). We also referred to [50], i.e., class and school characteristics are regulated by controlling fixed effects in the class.

In terms of family characteristics, such factors were controlled in variables, i.e., parents’ years of schooling, parents’ occupation (including farmers, self-employed and unemployed individuals, ordinary staff and workers, white-collar workers, corporate executives, party and government officials, and leaders of public institutions), and the family income status (the value ranging from one to five, the larger the value, the richer the family is). In addition, we also referred to the selection of control variables for family characteristics by [36], including the number of books and the status of computers and networks in the family (no computers = 0 with computers but no networks = 1 with computers and networks = 2). Since we focus on the peer effects among friends, the friends’ characteristics are controlled by adopting the fixed effect of friends as a variable.

### 3.3 Data source

The data are mainly derived from the 2013–2014 China Education Tracking Survey (CEPS), a nationally representative large-scale tracking survey project designed and implemented by the China Survey and Data Center of Renmin University of China. The 2013–2014 China Education Tracking Survey conducted the survey based on schools by taking the first grade (Grade 7) and the third grade (Grade 9) of junior middle school as the starting point, randomly selecting 28 county-level units (counties, districts, and cities) nationwide as survey points with the average years of schooling and the proportion of floating population as stratified variables. 112 schools and 438 classes were randomly selected from the selected county-level units for investigation. All students in the selected classes were included in the sample, and nearly 20,000 students were surveyed in the baseline survey.

In the CEPS questionnaire, when students claim someone as one of their five best friends, they need to provide detailed personal information, such as name, gender, household registration, contact information (mobile phone number and QQ number), and other characteristics, to ensure that the friend is real. Therefore, we select five of the students’ best friends as the research objects to obtain real information about their friends.

To make the data more credible, we proceed as follows: First, the samples exclude those whose original scores are above 150, whose standardized scores are negative, and those who do not have best friends. Second, the years of education are converted into the educational background of parents and the educational expectations of students and parents to achieve a unified approach.

To ensure robust research conclusions, we introduce the endogeneity issues and transmission mechanism into the 2014–2015 education tracking survey data and expand the cross-sectional data into panel data. However, due to the lack of new data samples, we mainly focus on this study’s 2013–2014 education tracking survey data. In the 2013–2014 survey, ninth graders were not available to obtain information due to junior high school graduation in 2014–2015, and a small portion of the samples of seventh graders could not be traced.

## 4 Empirical results and analyses

### 4.1 Benchmark regression

Table 2 reports the peer effects among students’ friends, that is, the impact of the number of friends with good grades on students’ performance. The first four columns respectively show the impact of each increase in the number of friends with good grades on the scores and average scores in Chinese, mathematics, and English, under the case of keeping other variables constant. Unlike the linear mean model that typically only controls for fixed effects in the class, we study the impact of changes in the proportion of students with good grades among friends and simultaneously control for fixed effects in both the class and friends.

**Table 2:** The impact of the number of friends with good grades on students’ performance.

	(1)	(2)	(3)	(4)
	Chinese score	Mathematics score	English score	Average score
Number of friends with good grades	0.478***	0.586***	0.509***	0.521***
	(0.0651)	(0.0683)	(0.0670)	(0.0565)
Gender	-4.204***	0.0570	-4.009***	-2.712***
	(0.506)	(0.485)	(0.518)	(0.429)
Cognitive ability	3.689***	5.112***	3.891***	4.232***
	(0.138)	(0.155)	(0.148)	(0.131)
Maternal education	0.0712*	0.0682*	0.105***	0.0808***
	(0.0365)	(0.0356)	(0.0361)	(0.0294)
Paternal education	0.172***	0.127***	0.172***	0.156***
	(0.0439)	(0.0402)	(0.0400)	(0.0355)
Number of books in the family	0.451***	0.215**	0.285***	0.311***
	(0.0899)	(0.0963)	(0.0889)	(0.0779)
Status of computers and networks	-0.234*	-0.527***	-0.350***	-0.365***
	(0.120)	(0.127)	(0.134)	(0.107)
Other control variables	Yes	Yes	Yes	Yes
Fixed effects in the class	Yes	Yes	Yes	Yes
Adjusted R^2^	0.246	0.242	0.272	0.304
N	10590	10582	10583	10558

Note:

*, **, and ***, respectively, indicate the significant coefficients at 10%, 5%, and 1%.

The numbers in the table represent the regression coefficients of the variables, and the numbers in parentheses present the robust standard deviations for clustering at the class level. (the same below).

Benchmark regression estimates show that, holding other variables constant, an increase in the number of friends with good grades will significantly improve students’ scores in Chinese, math, English and their average scores. Such an impact is more than twice the impact of the father’s years of schooling on individual scores, and more than four times that of the mother’s years of schooling on it. This indicates a significant positive correlation between the number of friends with good grades and students’ academic performance. Thus, the conclusion of the benchmark regression is consistent with hypothesis 1.

From the perspective of main control variables, compared with male students, female students perform better academically in all subjects, which is consistent with the descriptive statistical results and research conclusions of [24]. The scores of individual cognitive abilities tests have a significant positive impact on students’ academic performance in all subjects. It indicates that the stronger their ability, the better the academic performance, which is consistent with the conclusion of [51] on the impact of individual ability on academic performance and daily practice. Parents’ years of schooling have a significant positive effect on children’s academic performance, which may be because families with well-educated parents pay more attention to their children’s education and invest more in it. The number of books in a family also significantly improves students’ academic performance, especially in Chinese. It may be because more books in the family are conducive to students’ reading habits. However, owning a computer and network at home has a significant negative effect on students’ academic performance, possibly as it causes children to spend more time online and less time studying.

### 4.2 Endogeneity issues

#### 4.2.1 Instrumental variable approach

Although benchmark regression indicates that the number of friends with good grades could significantly improve students’ academic performance, there might be a self-selection issue that students with good grades are more inclined to choose to be friends with peers of the same excellent performance (correlation effect). The studies of [10, 52] both indicate that if the correlation effect caused by self-selection cannot be separated from endogenous effects, the estimated coefficients obtained by OLS regression may be inconsistent. However, endogeneity issues could also arise, when it is unable to exclude the influence of common factors (environmental effects) and non-observable friend characteristics (exogenous effects) on academic performance. In addition, since most of the students’ best friends are their peers in the same school or even class, there may be endogeneity issues caused by two-way causality.

The instrumental variable method is an important method to address endogeneity issues in identifying peer effects [45, 53]. Therefore, we select whether parents care about children’s choice of friends and their children’s hobbies as instrumental variables for the number of friends with good grades. The theoretical logic is that children’s choice of friends could be largely influenced by parents when they care about children’s choice of friends. Due to the general desire to have a successful child, parents tend to choose excellent peers as their children’s friends. When parents adopt a laissez-faire attitude towards the issue, children will make friends based on their own interests and hobbies. The issue of whether parents care about children’s choice of friends could affect children’s choices but not directly influence children’s academic performance. The regression results are shown in Table 3.

**Table 3:** Two-stage least squares estimation.

	(1)	(2)	(3)
	Student Grade Average	Stage 1	Stage 2
Whether parents care about children’s choice of friends	0.293	0.150***	
	(0.190)	(0.0322)	
Number of friends with good grades			2.209**
			(1.075)
Adjusted R^2^	0.297	0.110	0.230
N	10544	10796	10438
Statistics of Cragg-Donald Wald F			31.287

Note:

*, **, and *** indicate the significant coefficients at the 10%, 5%, and 1% levels, respectively.

The numbers in the table represent the regression coefficients of the variables, and the numbers in parentheses present the robust standard deviations for clustering at the class level.

From column 1 of Table 3, the effect of “Whether parents care about children’s choice of friends” on “student academic performance” is positive. Still, the result is insignificant, so the instrumental variable has no direct effect on student academic performance. According to the regression results in the first stage of Table 3, whether parents care about children’s choice of friends and what their children are interested in will significantly affect the number of children’s friends with good grades, which is consistent with the expectation of hypothesis 2 and fulfills the correlation requirement for instrumental variables. The results of the second-stage regression showed that making friends with good graders has a significant promoting effect on improving students’ average scores, and the regression coefficients obtained were higher than the estimated parameters of the benchmark regression. This indicates that the benchmark regression does not overestimate the impact of the number of friends with good grades on students’ academic performance. The important implication is that parents in impoverished families can improve their children’s friendships and academic performance by paying closer attention to children’s choice of friends and other aspects.

From the results of the weak instrumental variables test, it can be seen that the statistics of Cragg-Donald Wald F is 31.287, higher than the critical value of 16.38 at the 10% level specified by [54]. Thus, it is suggested that the instrumental variables chosen in this paper have strong explanatory power, and there is no problem with weak instrumental variables. Therefore, it is believed that the instrumental variables selected in this study have strong explanatory power and that there is no problem with weak instrumental variables. And it is credible for the results estimated using the two-stage least squares method. The increase in the number of friends with good grades does indeed improve students’ academic performance. The endogeneity issues caused by self-selection can be alleviated to a certain extent by using the instrumental variable method.

#### 4.2.2 Methods for panel data

Although characteristics variables such as parents’ years of education, occupation, and political status are controlled, it cannot be ruled out that the instrumental variables (whether parents care about children’s choice of friends) may affect children’s grades through other unobserved parental characteristics. For instance, the more parents concern themselves with their children’s friendships, the more they tend to focus on their children’s grades, subsequently impacting their academic performance. As a result, the externality of instrumental variables cannot be satisfied. Therefore, we perform cross-validation when using the panel data approach to ensure the robustness of the findings.

Although we can use two-period panel data, there is really no way to control for individual fixed effects because friend networks are highly stable once formed [17]. Obviously, best friends do not shift in the short term, and their grades are unlikely to rise or fall greatly in the short term. So, we cannot use this variation to identify the effect of peer effects among friends. In addition, we only have a two-period panel. Once controlling for individual fixed effects, it requires the inclusion of more than 10,000 dummy variables, which would be a significant loss of degrees of freedom. Important control variables like students’ gender, household registration and ethnicity, parents’ political affiliation, and education level do not change in the short run. Meanwhile, once individual fixed effects are controlled for, the effects of these variables are absorbed.

This paper utilizes both random and fixed effects in the panel data (see Table 4). In the random effects model, the results show that the peer effect has a significant positive effect on student academic performance. In addition, what we really want to control are fixed effects for friends, not individual fixed effects. 65% of friends are in the same class, and 81% are in the same school, so once we control for class fixed effects, we can control for most of the fixed effects among friends. For students whose best friends are not in the same school, we can partially control the fixed effects among friends by controlling for individual fixed effects. The regression results for both are shown in Table 4 below.

**Table 4:** Estimation of panel data.

	(1)	(2)
	random effects model	Controlling for partial individual fixed effects
Number of friends with good grades	1.000***	1.033***
	(0.128)	(0.130)
r2_a		0.476
N	11133	10920

Note:

*, **, and *** indicate the significant coefficients at the 10%, 5%, and 1% levels, respectively.

The numbers in the table represent the regression coefficients of the variables, and the numbers in parentheses present the robust standard deviations for clustering at the class level.

### 4.3 Quantile regression

As group behaviors and outcomes may not affect individuals symmetrically, it may be difficult to accurately estimate the magnitude and significance of peer effects using a simple linear mean model. To this end, we referred to the research method chosen by [55] and used quantile regression for analysis. Since the estimated coefficients obtained from quantile regression are expressed as the marginal effects of the explanatory variable on the explained variable at specific points, different quantiles can yield different quantile functions [39]. Therefore, it can distinguish the trend of changes for students with different performances under peer effect among friends.

In this study, we select five quantiles of 10, 30, 50, 70, and 90 for regression. If the increase of the quantile is not significantly changed along with the estimated coefficient of the number of friends with good grades, it indicates that the influence of peer effect on students’ performance is symmetrical. Otherwise, it is considered that the influence of peer effect among friends is asymmetric. Asymmetric situations can be further divided into two types. If the estimated coefficient could increase significantly with the quantile, it indicates that students with good grades are more affected by the peer effect among friends; if not, students with poorer grades could benefit more from having friends with better grades.

Results in the quantile regression demonstrate that the regression coefficients at all quantiles are significantly positive at the level of 1%. This implies that friends with good grades could help students with high or low grade averages, in line with the findings of [56, 57] regarding the impact of the academic performance of peers on that of students. Students with grades below 10 and above 90 were less affected by the peer effect among friends than students in the 30–70 quartile and above. This suggests that students with around median academic performance could gain more from their friends with good grades. This could be because similar cognitive abilities are required when learning from peers or in competitive learning environments [35] (Table 5). It is also consistent with [31] claims that high-achieving students benefit more from the peer effects. The only exception was a sudden drop in students above the 90th quantile in the peer effects among friends, probably because these students themselves had already scored highly and had relatively scant room to improve.

**Table 5:** Perform quantile regression based on average scores.

	(1)	(2)	(3)	(4)	(5)
	QR_10	QR_30	QR_50	QR_70	QR_90
Number of friends with good grades	0.498***	0.611***	0.606***	0.554***	0.403***
	(0.112)	(0.0820)	(0.0647)	(0.0596)	(0.0623)
Control variable	Yes	Yes	Yes	Yes	Yes
Fixed effect of Class	Yes	Yes	Yes	Yes	Yes
R^2^	0.213	0.216	0.216	0.215	0.198
N	11463	11463	11463	11463	11463

Note:

*, **, and *** indicate the significant coefficients at the 10%, 5%, and 1% levels, respectively.

The numbers in the table represent the regression coefficients of the variables, and the numbers in parentheses present the robust standard deviations for clustering at the class level.

### 4.4 Heterogenous treatment effects

Peer effects among friends vary with gender, household registration, and academic success. [23, 24] found that raising the proportion of female students in a class or grade could considerably improve both males’ and females’ academic performance. A higher proportion of female students could help to improve classroom discipline and learning environment, and thus, teachers can be less fatigued and focus on imparting knowledge.). According to [58], favorable settings more positively impact female students, and male students are more vulnerable to negative situations. Besides, there would be a large influx of immigrants into cities in the process of urbanization. Although cities centralize high-quality educational resources, such resources are allocated according to local household registration. Students with different household registrations may perform differently in terms of their learning, as those with local household registrations have access to more public educational resources. In 1986, the Law of the People’s Republic of China on Compulsory Education entrusted the county-level or district-level government with the responsibility of raising funds for compulsory education. Therefore, whether students have local registered residences is related to how much local public education services they can enjoy). Thus, group regression was carried out based on gender and household registration, and the coefficient differences were examined between various groups.

Regression results for columns (1) and (2) in Table 6 indicate that both male and female students could benefit from friends with good grades, and the number of friends with good grades has a greater impact on female students than male students. The reason could be that female students gain more from a group of high-achieving friends since they are generally more disciplined and less susceptible to negative influences. The group regression results are in accordance with those of [23, 58, 59].

**Table 6:** Average scores by gender and registered registration subjected to group regression.

	(1)	(2)	(3)	(4)
	Female students	Male students	Non-local registered residence	Local registered residence
Number of friends with good grades	0.558***	0.414***	0.465***	0.519***
	(0.0683)	(0.0875)	(0.165)	(0.0630)
Control variable	Yes	Yes	Yes	Yes
Fixed effect of Class	Yes	Yes	Yes	Yes
Adjusted R2	0.283	0.270	0.298	0.306
N	5255	5303	1646	8912

Note:

*, **, and *** indicate the significant coefficients at the 10%, 5%, and 1% levels, respectively.

The numbers in the table represent the regression coefficients of the variables, and the numbers in parentheses present the robust standard deviations for clustering at the class level.

All students could benefit from friends with good grades, but the number of friends with good grades benefits students with local household registration more significantly, according to the results of group regression on students’ household registration. This is in line with the findings of [49] on how neighborhood surroundings affected students with different household registration in academic performance. Also, due to the hindrances on household registration, some schools do not accept students with non-local household registration while those willing to accept are in a relatively poor environment, which could account for the relatively smaller impact of the peer effects among friends. Besides, students with local household registration may achieve relatively better academic performance. According to quantile regression, students with better grades are more influenced by their academically successful friends.

## 5 Further discussion

### 5.1 Transmission mechanism

This study focuses on the impact of social interaction among friends on individuals’ learning preferences and behaviors due to the insufficient material on information transmission and knowledge sharing in this context. According to [60] social structure theory, the influence of peers on individuals’ preferences and behaviors is primarily realized through the normative and comparative functions. The normative function implies that the individual could be infected by the emotions and beliefs in the group, leading them to comply and take actions consistent with the group. In contrast, the comparison function refers to an individual’s ability to revise their own evaluation based on the objective state of group members as a benchmark [45]. Thus, we suggest that parents’ and students’ educational expectations and input are key factors in realizing the peer effects among friends. For this, the transmission mechanism of the peer effect is described by using the two-way fixed effect model of panel data in this work.

#### 5.1.1 Compliance effect

The compliance effect describes how individuals adjust their own behavior to match that of group members on shifts in the group’s features. Social interactions among friends have the potential to sway an individual’s preferences and behaviors by those of group, as students tend to imitate each other’s behavior, which makes the group members more likely to exhibit consistent preferences and behaviors. The compliance effect is precisely used to describe the beneficial impact of groups on individuals. For instance, [60] viewed the compliance effect as a crucial key to the upholding of group norms, while [61] described how groups can affect individual preferences and behaviors in the status and compliance model.

Group identification with learning would improve with increased number of friends who excel academically. Students would raise their educational expectations to adjust to environmental changes in the group and spend more time and energy on learning with less class truancy and absenteeism, which could enhance their academic performance. With the increasing number of high-achieving friends, students with good grades could be under pressure to do better and compete with their peers. In contrast, those with average grades or below would devote more time and effort to their studies to reach the average level, as shown in the quantile regression. Looking at the overall picture, all students could benefit from their friends who perform well academically.

From the regression analysis in Table 7, students would hold higher expectations for education with an increase in high-achieving friends. They spend more time on learning rather than entertainment with less class truancy and absenteeism. Students’ educational expectations have a significant positive impact on their academic performance [45, 49]. Also, it is not difficult to infer from daily practice that students’ academic performance could be improved by more time spent learning and less on class truancy and absenteeism, which is consistent with the benchmark regression. Thus, hypothesis 3a is valid, i.e., a compliance effect exists.

**Table 7:** Compliance effects.

	(1)	(2)	(3)	(4)
	Students’ educational expectations	Study and entertainment time ratio	Frequency of class lateness	Frequency of class absenteeism
Number of friends with good grades	0.282***	0.0347***	-0.0219***	-0.00648**
	(0.0235)	(0.0120)	(0.00508)	(0.00327)
Control variable	Yes	Yes	Yes	Yes
Fixed effect of Class	Yes	Yes	Yes	Yes
Adjusted R2	0.220	0.175	0.0630	0.0279
N	10799	8308	10797	10791

Note:

*, **, and *** indicate the significant coefficients at the 10%, 5%, and 1% levels, respectively.

The numbers in the table represent the regression coefficients of the variables, and the numbers in parentheses present the robust standard deviations for clustering at the class level.

#### 5.1.2 Anchoring effect

The anchoring effect describes how parents evaluate their children’s academic performance by taking children’s friends as a frame of reference, thus, to adjust their educational expectations and inputs in their children. Drawing on the anchoring effect in psychology, it demonstrates that the chosen reference point largely determines an individual’s evaluation of something. Parents would also consider that of their children’s friends when evaluating children’s academic performance. Parents would raise reference standards in tandem with the increasing number of children’s high-achieving friends, thus holding higher educational expectations and performance requirements for their children. Parents would devote more time and effort to their children’s education, such as checking homework and tutoring them frequently, so that their children are on par with their friends in academic performance, coupled with the desire for their children’s success. (Note: Considering that parents need to have a higher level of education to tutor their children in middle school studies, the samples of parents whose years of schooling are over ten years.

The regression results in Table 8 demonstrate that parents’ educational expectations and grade requirements for their children will rise in tandem with the increasing number of children’s high-achieving friends. Parents would devote more time and effort to their children’s education, such as checking homework and tutoring them frequently, so that their children live up to their expectations or be on par with their friends in academic performance. All these results are consistent with hypothesis 3b, indicating the possibility of the anchoring effect. [49] demonstrated that parents’ greater educational expectations and grade requirements for their children could enhance children’s academic performance. Parents who tutor and check homework more frequently could also help their children perform better academically.

**Table 8:** Anchoring effect.

	(1)	(2)	(3)	(4)
	Parents’ educational expectation for children	Parents’ academic requirements for children	Frequency of checking homework	Frequency of tutoring
Number of friends with good grades	0.173***	0.0611***	0.0231***	0.0337**
	(0.0222)	(0.00678)	(0.00859)	(0.0138)
Control variable	Yes	Yes	Yes	Yes
Fixed effect of Class	Yes	Yes	Yes	Yes
Adjusted R2	0.204	0.134	0.193	0.160
N	10782	10774	10758	5377

Note:

*, **, and *** indicate the significant coefficients at the 10%, 5%, and 1% levels, respectively.

The numbers in the table represent the regression coefficients of the variables, and the numbers in parentheses present the robust standard deviations for clustering at the class level.

### 5.2 Robustness test

The assessment criteria of academic achievement need to be modified to prevent estimation bias by measurement errors, though the two-way fixed-effect model of panel data and the instrumental variable method have been previously used to mitigate potential endogeneity issues in this study. The dimensionless standardized score with a mean value of 0 and a standard deviation of 1 could be obtained by deducting the mean value of the original score and then dividing the result by the standard deviation, results as presented in Table 9.

**Table 9:** Robustness test 1.

	(1)	(2)	(3)	(4)
	Standardized Chinese score	Standardized Mathematics score	Standardized English score	Standardized average score
Number of friends with good grades	0.0485***	0.0593***	0.0515***	0.0605***
	(0.00661)	(0.00691)	(0.00677)	(0.00657)
Control variable	Yes	Yes	Yes	Yes
Fixed effect of Class	Yes	Yes	Yes	Yes
Adjusted R2	0.246	0.242	0.272	0.304
N	10590	10582	10583	10558

Note:

*, ** and *** respectively indicate the significant coefficients at the level of 10%, 5% and 1%.

The numbers in the table represent the regression coefficients of the variables, and the numbers in parentheses present the robust standard deviations for clustering at the class level.

As the robustness test shows, there could still be a considerable beneficial impact of the number of friends with good grades on students’ academic performance. Every increase of friends with good grades could result respectively in an increase of 0.0396, 0.0466, 0.0402, and 0.0479 standard deviations in students’ Chinese, Mathematics, English, and average scores, assuming all other variables hold the same. All these increases are significant at the level of 1% and consistent with the regression results of benchmark regression and instrumental variable method. This indicates the robustness of benchmark regression results in the study.

In addition to replacing the explained variables, the original value on the number of friends with good grades in the questionnaire was adopted to test the benchmark regression (where "0" indicates "no high-achieving friends", and "1" indicates "one or two high-achieving friends", "2" indicates "many high-achieving friends"), in order to prevent subjective estimation bias by the average of the number of high-achieving friends in the data processing. The regression results are shown in Table 10.

**Table 10:** Robustness test 2.

	(1)	(2)	(3)	(4)
	Chinese score	Mathematics score	English score	Average score
Number of friends with good grades	1.208***	1.386***	1.196***	1.257***
	(0.158)	(0.158)	(0.157)	(0.134)
Control variable	Yes	Yes	Yes	Yes
Fixed effect of Class	Yes	Yes	Yes	Yes
Adjusted R2	0.247	0.243	0.272	0.305
N	10590	10582	10583	10558

Note:

*, ** and *** respectively indicate the significant coefficients at the level of 10%, 5% and 1%.

The numbers in the table represent the regression coefficients of the variables, and the numbers in parentheses present the robust standard deviations for clustering at the class level.

From the robustness test in Table 10, students’ academic performance could still be greatly affected by more friends with good grades after the original value being applied. Consequently, the result in the benchmark regression is robust. This indicates that peer effects could occur among friends, even if the regression coefficient obtained at this point has little marginal significance.

## 6 Conclusions

Using data from the China Education Tracking Survey, we examine the impact of academically successful friends on student performance. The study found that students significantly improved their academic performance as the number of their friends with good grades increased. The two-stage least square approach (2SLS) was used to assess the peer effect among friends to ensure a robust conclusion, the results of which are essentially compatible with the conclusion in the benchmark regression. Meanwhile, the heterogeneity of different groups is also examined in terms of gender, average performance, and household registration of students. We found that peer effects are more easily exerted among female students and those with better average grades or local household registration. And all students can benefit from friends with good grades, no matter how their grades are, whether they are male or female students, or whether they have local household registration.

Our study also examines the transmission mechanism of peer effects among friends. It indicates that the attitude about education would change in the group as the number of friends with good marks rises. Students will hold higher educational expectations and devote more time and attention to their learning to adjust themselves to shifts in the learning environment in groups. Moreover, parents could evaluate their children’s academic performance by taking their children’s friends as a frame of reference. They would raise higher reference standards in tandem with the increasing number of children’s high-achieving friends, holding higher educational expectations and performance requirements for their children, thus devoting more time and effort to children’s education. All these would help students perform better academically. The results in this study are consistent with the research conclusions of [17], which found that the desk-mate with good grades could be conducive to individuals’ academic performance, and an outgoing and approachable desk-mate could contribute to the development of students’ non-cognitive abilities. The findings in this study are also consistent with the conclusions of [6, 24], in which the former suggested that a higher proportion of female (or more disciplined) students could help improve students’ performance, while the latter found that an additional performance improvement of 15%-18% could be reached by allowing individuals to freely choose their peers compared to randomly choosing peers.

As the ancient Chinese proverb goes, "lies down with dogs must rise up with fleas", which means that one could be influenced by close association. The best friends of most students could be their peers in the school or even class, thereby attending a prestigious school or key class is indeed beneficial to making friends with better marks, which would promote their academic performance. However, the competition in basic education is not entirely irrational, as students from poor families are disadvantaged when it comes to making friends with better academic performance. Besides, the study also found that students’ choice of friends is largely affected by parents’ attitudes towards this issue. And the peer effects among friends would be partly realized by the time and energy parents invested in their children’s education. Therefore, for families of lower socio-economic status, focusing more on their children’s social interactions with friends presents a viable alternative to enhance academic performance. Therefore, for families with poor economic status, it’s a feasible alternative to improve children’s academic performance by paying more attention to their children’s social interaction with friends.

## Supporting information

S1 Appendix Effect of compliance effects on student academic performance. (DOCX)

S2 Appendix Impact of anchoring effects on student academic performance. (DOCX)

S1 Data (ZIP)

S2 Data (ZIP)

S3 Data (ZIP)

S4 Data (ZIP)
