# Supplementary material for: Peer effects among friends on students’ cognitive abilities: An analysis based on emotional distance
Source: PLoS One. 2025 Feb 3;20(2):e0312190. doi: 10.1371/journal.pone.0312190 (PMC11790103; doi:10.1371/journal.pone.0312190)
Supplement: S1 Appendix — (DOCX) [file pone.0312190.s001.docx]

Exhibit 1 Effect of Compliance Effects on Student academic performance

|  | (1) | (2) | (3) | (4) |
| --- | --- | --- | --- | --- |
|  | Grade Average | Grade Average | Grade Average | Grade Average |
| Students' educational expectations | 0.685^***^ |  |  |  |
|  | (0.0281) |  |  |  |
| Study and entertainment time ratio |  | 0.223^***^ |  |  |
|  |  | (0.0541) |  |  |
| Frequency of class lateness |  |  | -0.790^***^ |  |
|  |  |  | (0.141) |  |
| Frequency of class absenteeism |  |  |  | -1.470^***^ |
|  |  |  |  | (0.220) |
| Control variable | Yes | Yes | Yes | Yes |
| Fixed effect of Class | Yes | Yes | Yes | Yes |
| Adjusted R2 | 0.362 | 0.297 | 0.300 | 0.301 |
| N | 10546 | 8113 | 10545 | 10539 |

Note: Regression equations were used to test the compliance effect. The regression results in Exhibit 1 show that the compliance effect significantly impacts student academic performance. In other words, students' educational expectations and the ratio of study to recreation time significantly increased their academic performance, while the frequency of lateness and absenteeism significantly decreased it.
