# Supplementary material for: Peer effects among friends on students’ cognitive abilities: An analysis based on emotional distance
Source: PLoS One. 2025 Feb 3;20(2):e0312190. doi: 10.1371/journal.pone.0312190 (PMC11790103; doi:10.1371/journal.pone.0312190)
Supplement: S2 Appendix — (DOCX) [file pone.0312190.s002.docx]

Exhibit 2 Impact of Anchoring Effects on Student Academic Performance

|  | (1) | (2) | (3) | (4) |
| --- | --- | --- | --- | --- |
|  | Grade Average | Grade Average | Grade Average | Grade Average |
| Parents' educational expectation for children | 0.659*** |  |  |  |
|  | (0.0316) |  |  |  |
| Parents' academic requirements for children |  | 3.342*** |  |  |
|  |  | (0.106) |  |  |
| Frequency of checking homework |  |  | -0.567*** |  |
|  |  |  | (0.0646) |  |
| Frequency of tutoring |  |  |  | -0.627*** |
|  |  |  |  | (0.0727) |
| Control variable | Yes | Yes | Yes | Yes |
| Fixed effect of Class | Yes | Yes | Yes | Yes |
| Adjusted R2 | 0.349 | 0.413 | 0.302 | 0.302 |
| N | 10529 | 10521 | 10508 | 10431 |

Note: Anchoring effects were tested using regression equations. From the regression results in Exhibit 2, it is clear that anchoring effects have a significant impact on student achievement. Parents' educational expectations and academic demands on their children significantly increase student achievement, while the frequency of parental checking of homework or tutoring has a significant positive impact on student achievement.
