# Supplementary material for: Peer effects among friends on students’ cognitive abilities: An analysis based on emotional distance
Source: PLoS One. 2025 Feb 3;20(2):e0312190. doi: 10.1371/journal.pone.0312190 (PMC11790103; doi:10.1371/journal.pone.0312190)
Supplement: S1 Data — (ZIP) [file pone.0312190.s003.zip › myfile_c.rtf]

	(1)	(2)	(3)	(4)	
	stdas	stdas	stdas	stdas	
edu1	0.685***				
	(0.0281)				
t1		0.223***			
		(0.0541)			
late			-0.790***		
			(0.141)		
absent				-1.470***	
				(0.220)	
r2_a	0.362	0.297	0.300	0.301	
N	10546	8113	10545	10539	
Standard errors in parentheses
* p < 0.1, ** p < 0.05, *** p < 0.01
