# Supplementary material for: Peer effects among friends on students’ cognitive abilities: An analysis based on emotional distance
Source: PLoS One. 2025 Feb 3;20(2):e0312190. doi: 10.1371/journal.pone.0312190 (PMC11790103; doi:10.1371/journal.pone.0312190)
Supplement: S1 Data — (ZIP) [file pone.0312190.s003.zip › myfile_d.rtf]

	(1)	(2)	(3)	(4)	
	stdas	stdas	stdas	stdas	
edu2	0.659***				
	(0.0316)				
pes		3.342***			
		(0.106)			
prs			-0.567***		
			(0.0646)		
prp				-0.627***	
				(0.0727)	
r2_a	0.349	0.413	0.302	0.302	
N	10529	10521	10508	10431	
Standard errors in parentheses
* p < 0.1, ** p < 0.05, *** p < 0.01
