# Supplementary material for: Peer effects among friends on students’ cognitive abilities: An analysis based on emotional distance
Source: PLoS One. 2025 Feb 3;20(2):e0312190. doi: 10.1371/journal.pone.0312190 (PMC11790103; doi:10.1371/journal.pone.0312190)
Supplement: S1 Data — (ZIP) [file pone.0312190.s003.zip › myfile_e.rtf]

	(1)	(2)	
	ptime	ecost	
fec	0.0174	-33.48	
	(0.0237)	(29.20)	
r2_a	0.0835	0.181	
N	10029	10093	
Standard errors in parentheses
* p < 0.1, ** p < 0.05, *** p < 0.01
