# Supplementary material for: Peer effects among friends on students’ cognitive abilities: An analysis based on emotional distance
Source: PLoS One. 2025 Feb 3;20(2):e0312190. doi: 10.1371/journal.pone.0312190 (PMC11790103; doi:10.1371/journal.pone.0312190)
Supplement: S1 Data — (ZIP) [file pone.0312190.s003.zip › myfile2.rtf]

	(1)	(2)	(3)	(4)	
	stdchn	stdmat	stdeng	stdas	
fec_a	1.208***	1.386***	1.196***	1.257***	
	(0.158)	(0.158)	(0.157)	(0.134)	
r2_a	0.247	0.243	0.272	0.305	
N	10590	10582	10583	10558	
Standard errors in parentheses
* p < 0.1, ** p < 0.05, *** p < 0.01
