# Supplementary material for: Peer effects among friends on students’ cognitive abilities: An analysis based on emotional distance
Source: PLoS One. 2025 Feb 3;20(2):e0312190. doi: 10.1371/journal.pone.0312190 (PMC11790103; doi:10.1371/journal.pone.0312190)
Supplement: S1 Data — (ZIP) [file pone.0312190.s003.zip › myfile4.rtf]

	(1)	(2)	
	stdas	stdas	
fec	1.000***	1.033***	
	(0.128)	(0.130)	
r2_a		0.476	
N	11133	10920	
Standard errors in parentheses
* p < 0.1, ** p < 0.05, *** p < 0.01
