# Supplementary material for: Peer effects among friends on students’ cognitive abilities: An analysis based on emotional distance
Source: PLoS One. 2025 Feb 3;20(2):e0312190. doi: 10.1371/journal.pone.0312190 (PMC11790103; doi:10.1371/journal.pone.0312190)
Supplement: S1 Data — (ZIP) [file pone.0312190.s003.zip › myfile5.rtf]

	(1)	(2)	(3)	(4)	(5)	
	stdas	stdas	stdas	stdas	stdas	
fec	0.498***	0.611***	0.606***	0.554***	0.403***	
	(0.112)	(0.0820)	(0.0647)	(0.0596)	(0.0623)	
r2	0.213	0.216	0.216	0.215	0.198	
N	11463	11463	11463	11463	11463	
Standard errors in parentheses
* p < 0.1, ** p < 0.05, *** p < 0.01
