# Supplementary material for: Peer effects among friends on students’ cognitive abilities: An analysis based on emotional distance
Source: PLoS One. 2025 Feb 3;20(2):e0312190. doi: 10.1371/journal.pone.0312190 (PMC11790103; doi:10.1371/journal.pone.0312190)
Supplement: S1 Data — (ZIP) [file pone.0312190.s003.zip › temp1_a.docx]

Summary Statistics

| VarName | Obs | Mean | SD |
| --- | --- | --- | --- |
| stdchn | 17970 | 70.05 | 9.849 |
| stdmat | 17958 | 70.03 | 9.886 |
| stdeng | 17962 | 70.05 | 9.889 |
| stdas | 17917 | 70.06 | 8.610 |
| fec | 18321 | 2.58 | 1.398 |
| age | 18048 | 13.52 | 1.242 |
| gender | 18168 | 0.52 | 0.500 |
| location | 18107 | 0.82 | 0.384 |
| hukou | 17554 | 0.31 | 0.463 |
| nation | 18382 | 0.91 | 0.282 |
| onec | 18422 | 0.43 | 0.495 |
| sib | 15706 | 0.64 | 1.020 |
| hm | 18154 | 0.86 | 0.344 |
| hf | 18154 | 0.81 | 0.396 |
| health | 18274 | 4.06 | 0.895 |
| hospital | 18291 | 0.08 | 0.274 |
| medu | 18091 | 9.52 | 3.548 |
| fedu | 18055 | 10.30 | 3.130 |
| mwork1 | 16971 | 0.55 | 0.497 |
| mwork2 | 16971 | 0.26 | 0.440 |
| mwork3 | 16971 | 0.10 | 0.303 |
| mwork4 | 16971 | 0.08 | 0.278 |
| fwork1 | 17047 | 0.45 | 0.498 |
| fwork2 | 17047 | 0.17 | 0.374 |
| fwork3 | 17047 | 0.25 | 0.432 |
| fwork4 | 17047 | 0.13 | 0.337 |
| mpolc | 18427 | 0.04 | 0.195 |
| fpolc | 18427 | 0.06 | 0.231 |
| pgb | 18054 | 0.82 | 0.262 |
| relation | 17991 | 0.84 | 0.369 |
| inth | 18353 | 0.87 | 0.335 |
| ninc | 17681 | 2.99 | 0.555 |
| book | 18377 | 3.15 | 1.207 |
| computer | 18205 | 1.29 | 0.911 |
| edu1 | 18302 | 16.30 | 3.489 |
| t1 | 13966 | 0.79 | 1.470 |
| late | 18312 | 1.25 | 0.619 |
| absent | 18302 | 1.09 | 0.431 |
| edu2 | 18289 | 15.81 | 3.205 |
| pes | 18324 | 2.83 | 0.906 |
| prs | 18238 | 1.40 | 1.175 |
| prp | 18065 | 1.02 | 1.112 |
