# Supplementary material for: Peer effects among friends on students’ cognitive abilities: An analysis based on emotional distance
Source: PLoS One. 2025 Feb 3;20(2):e0312190. doi: 10.1371/journal.pone.0312190 (PMC11790103; doi:10.1371/journal.pone.0312190)
Supplement: S1 Data — (ZIP) [file pone.0312190.s003.zip › temp2_a.docx]

Summary Statistics

| VarName | Obs | Mean | SD |
| --- | --- | --- | --- |
| stdchn | 17970 | 70.05 | 9.849 |
| stdmat | 17958 | 70.03 | 9.886 |
| stdeng | 17962 | 70.05 | 9.889 |
| fec | 18321 | 2.58 | 1.398 |
| age | 18048 | 13.52 | 1.242 |
| gender | 18168 | 0.52 | 0.500 |
| rbt | 18427 | -0.00 | 0.862 |
| sib | 15706 | 0.64 | 1.020 |
| nation | 18382 | 0.91 | 0.282 |
| location | 18107 | 0.82 | 0.384 |
| hukou | 17554 | 0.31 | 0.463 |
| hm | 18154 | 0.86 | 0.344 |
| hf | 18154 | 0.81 | 0.396 |
| onec | 18422 | 0.43 | 0.495 |
| health | 18274 | 4.06 | 0.895 |
| hospital | 18291 | 0.08 | 0.274 |
| inth | 18353 | 0.87 | 0.335 |
| medu | 18091 | 9.52 | 3.548 |
| fedu | 18055 | 10.30 | 3.130 |
| mpolc | 18427 | 0.04 | 0.195 |
| fpolc | 18427 | 0.06 | 0.231 |
| ninc | 17681 | 2.99 | 0.555 |
| relation | 17991 | 0.84 | 0.369 |
| book | 18377 | 3.15 | 1.207 |
| computer | 18205 | 1.29 | 0.911 |
| fged | 16853 | 0.50 | 0.455 |
| flot | 16669 | 0.83 | 0.292 |
| frc | 16814 | 0.65 | 0.336 |
| frsnc | 16816 | 0.17 | 0.247 |
