# Supplementary material for: Peer effects among friends on students’ cognitive abilities: An analysis based on emotional distance
Source: PLoS One. 2025 Feb 3;20(2):e0312190. doi: 10.1371/journal.pone.0312190 (PMC11790103; doi:10.1371/journal.pone.0312190)
Supplement: S1 Data — (ZIP) [file pone.0312190.s003.zip › myfile.rtf]

	(1)	(2)	(3)	
	stdas	fec	stdas	
pgf	0.293	0.150***		
	(0.190)	(0.0322)		
fec			2.209**	
			(1.075)	
r2	0.328	0.148	0.264	
r2_a	0.297	0.110	0.230	
N	10544	10796	10438	
F	.	.	65.30	
Standard errors in parentheses
* p < 0.1, ** p < 0.05, *** p < 0.01
